# Chronological Changes in Sagittal Femoral Bowing after Primary Cementless Total Hip Arthroplasty: A Comparative 3D CT Study

**DOI:** 10.3390/jpm13121704

**Published:** 2023-12-13

**Authors:** Francesco Pardo, Antonino Amedeo La Mattina, Emanuele Diquattro, Stefano Lucchini, Marco Viceconti, Andrea Minerba, Francesco Castagnini, Francesco Traina

**Affiliations:** 1 Orthopedics-Traumatology and Prosthetic Surgery and Hip and Knee Revision, IRCCS Istituto Ortopedico Rizzoli, 40136 Bologna, Italy; 2 Medical Technology Lab, IRCCS Istituto Ortopedico Rizzoli, 40136 Bologna, Italy; 3 Department of Industrial Engineering, Alma Mater Studiorum—University of Bologna (IT), 40136 Bologna, Italy; 4 Department of Biomedical and Neuromotor Science-DIBINEM, University of Bologna, 40127 Bologna, Italy; francesco.traina@ior.it; 5 Head of Ortopedia-Traumatologia e Chirurgia Protesica e dei Reimpianti d’Anca e di Ginocchio, IRCCS Istituto Ortopedico Rizzoli, Via Pupilli 1, 40136 Bologna, Italy

**Keywords:** procurvatum, anatomic stem, bowing, modification, deformation, stem alignment, impingement, revision, total hip arthroplasty

## Abstract

Little is known about dynamic changes of femoral anatomy after total hip arthroplasty (THA), in particular about sagittal femoral bowing (SFB). A 3D CT study was designed to evaluate the chronological changes of SFB after cementless femoral stem implantation for primary THA. Ten patients who underwent unilateral primary THA with a cementless femoral stem, with 2 consecutive CT scans (extending from the fourth lumbar vertebra to the tibial plateaus), performed before THA and at least 3 years after THA, were enrolled. The 3D models of femurs were created using image segmentation software. Using the two CT scans, SFB values of the proximal and middle thirds were calculated on the replaced and untreated sides by two different observers. Eight anatomical stems and two conical stems were involved. The post-operative CT was performed at an average follow-up of 6.5 years after THA (range: 3–12.5). The measurements performed by the two observers did not differ in the proximal and middle regions. A significant difference between the pre-operative and post-operative SFB compared to the untreated side was found in the proximal femur segment (*p* = 0.004). Use of a cementless stem in THA induced chronological changes in SFB of the proximal femur, after a minimum timespan of 3 years.

## 1. Introduction

The femur has three-dimensional geometry in which a double bowing on the sagittal and coronal axis can be appreciated [1,2]. In a Japanese population, the total bowing was reported to be around 9°, with sagittal bowing accounting for 8.7° and coronal bowing for only 0.1° [1]. Femoral bowing, as well as femoral morphology, can be influenced by many factors: age, sex, ethnicity, femoral length, and density [3]. In particular, the femur does not remain static with age: in women (who show a more curved femur), bowing increases with age [3,4]. This structural chronological change is not without practical consequences. Femoral bowing increases tensile stress, which in turn increases femoral bowing, leading to a vicious circle of insufficiency fractures, as well as possible premature wear of adjacent joints, especially if other whistling or predisposing factors are present [5].

Moreover, some commercially available intramedullary nails may not match with severely bowed femurs, representative of up to 11.5% of the Korean population [6,7]. Similarly, femoral bowing influences the position of the intramedullary guide rod during total knee arthroplasty and revision surgery. This can lead to more flexed femoral components and axial malalignment, leading to a greater risk of implant failure or pain resulting from malposition of the implant [8]. In total hip arthroplasty (THA), femoral bowing was reported to influence the prosthetic range of motion even in cases of correct combined anteversion [9]. On the other side, the influence of cementless primary stems in THA on the chronological changes induced on sagittal femoral bowing (SFB) has been overlooked. Possible chronological modifications of SFB after THA may be of practical interest for primary component positioning, or in cases of stem revision or ipsilateral total knee arthroplasty, on which accurate pre-operative planning can be based.

Thus, a retrospective study on consecutive CT scans performed before and after THA was designed, using the contralateral untreated side as a control group. Our study aimed to evaluate the dynamic SFB changes induced by cementless primary femoral stems, and to compare these variations to the chronological changes occurring on the untreated side. We hypothesized that SFB could be significantly increased after stem implantation.

## 2. Materials and Methods

### 2.1. Patient Cohort Selection

The study was approved by the local ethics committee (CE-AVEC 349/2021/Oss/IOR, 10 May 2021). All procedures performed in this study comply with the ethical standards of the Institutional Research Committee and the 1964 Helsinki Declaration and its subsequent amendments or comparable ethical standards.

The CT scan database of a tertiary center, from January 2005 to April 2019, was screened. All patients who underwent at least 2 consecutive CT scans of the hip and pelvis, extending from the fourth lumbar vertebra to the tibial plateaus, were selected. The first CT scan included native joints without metal hardware or arthroplasty. The second CT scan was performed at least three years after THA, to evaluate the consolidated situation after THA.

The exclusion criteria were as follows: patients with fractures of the screened bone segments during the period between the 2 CTs; patients with contralateral hip and homolateral or contralateral knee arthroplasty; the presence of metal hardware; and patients who underwent procedures other than primary cementless THA. Patients with reversed radius of curvature (due to high femoral dysmorphism) of at least one of the two sides, and patients whose femoral segment length from the inferior border of the lesser trochanter to the transepicondylar axis was <35 cm or >40 cm, were also excluded from the study population, as these outliers would not be eligible for measurement.

The pre- and post-surgery SFB of the THA side was evaluated and compared in the two consecutive CT scans, in each case. The same evaluations were performed on the contralateral untreated femur, acting as a control group, in order to exclude alterations due to aging.

Demographics were collected. The anatomical features of the native hip and femur (treated and untreated sides) were assessed according to the measurement techniques described in other papers [10,11]; THA component positioning was also assessed for the THA side [10,11].

### 2.2. Calculation of Femur Curvature

The method used for the assessment of SFB was derived from the work of Abdelal et al. [9], with some modifications regarding femoral canal centroid calculation, as described below. Two observers performed the measurements to check for accuracy and repeatability.

**Figure 1:**
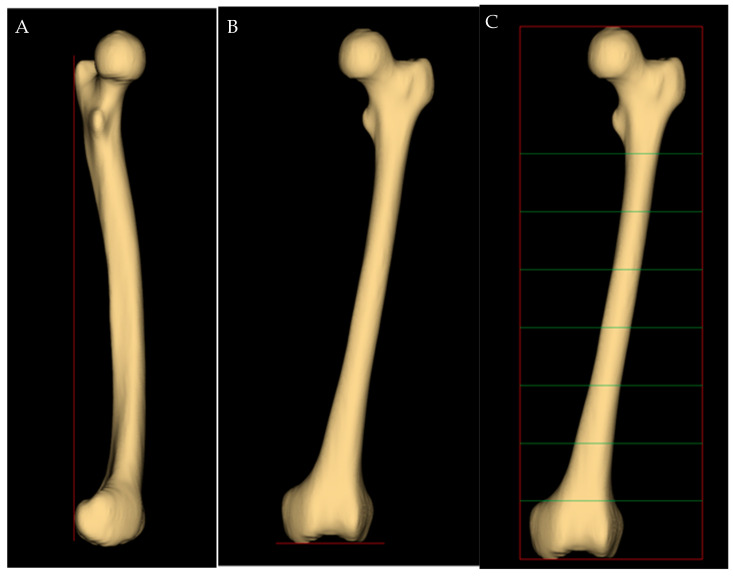
(**A**) Side view. Both condyles overlapped, and a vertical plane (red line) is tangent to the posterior intercondylar line and the posterior margin of the greater trochanter. (**B**) Anteroposterior view obtained by rotating the image (**A**) by 90° around the vertical axis, showing the reference axial plane. (**C**) Anteroposterior view of the femur, showing the axial planes at which femur canal was segmented (in green).

Image segmentation was performed using the free and open-source software 3D Slicer^®^ (version 4.11.20210226, Brigham and Women’s Hospital, Boston, MA, USA). Before the measurements, the femurs were three-dimensionally oriented in a standard configuration, so that a plane was simultaneously tangent to the most posterior portion of the femoral condyles and the posterior margin of the greater trochanter (Figure 1A). The reference axial plane was placed at the lowest margin of the two condyles (Figure 1B), and the femur canal was then manually segmented at seven axial planes with 5 cm steps (Figure 1C).

**Figure 2:**
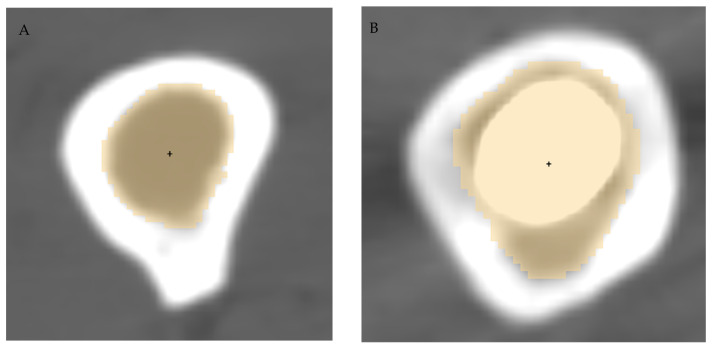
Segmentation of the femur canal (yellow shading) and identification of canal centroid (black dot) in the native side (**A**) and in the prosthetic side (**B**).

The radius of curvature was calculated at three levels of the femur, namely 10 (distal), 20 (middle) and 30 (proximal) cm above the reference axial plane, as well as for the whole femur. To measure the center of the medullary canal at each level, the level tracing method was used on the inner side of the medullary canal, with possible manual cleaning. Canal centroid was calculated as the geometric center of the level segmentation (Figure 2) using Matlab^®^ (version R2019b, The MathWorks Inc, Natick, MA, USA).

**Figure 3:**
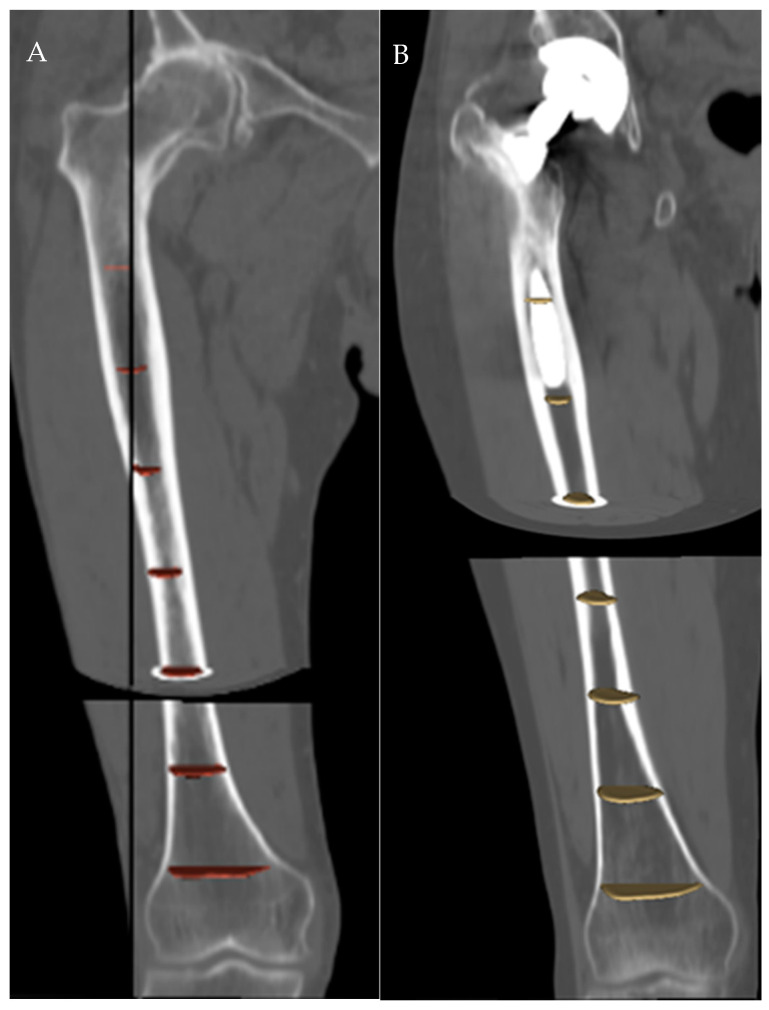
Segmentation of the femoral canal in a native (**A**) and a prosthetic (**B**) femur.

It is known from geometry that only one circle passes through three non-aligned points, so for the distal third of the femur, we used the centroids of the slices at 5 (point 1), 10 (point 2) and 15 (point 3) cm above the reference axial plane; for the middle third, those at 15, 20 and 25 cm; and for the proximal third, those at 25, 30 and 35 cm, respectively. For the whole femur, centroids at 5, 20, and 35 cm were used. (Figure 3).

The following Formula (1a) and (1b) was used to calculate the coordinates of the centers of the circles:

(1a) $$\;\;\;\;x_{C}=\frac{m_{1}m_{2}\left(y_{1}-y_{3}\right)+m_{2}\left(x_{1}+x_{2}\right)-m_{1}\left(x_{2}+x_{3}\right)}{2\left(m_{2}-m_{1}\right)}$$

(1b) $${\;y}_{C}=\frac{m_{2}\left(y_{2}+y_{3}\right)-m_{1}\left(y_{1}+y_{2}\right)-\left(x_{1}-x_{3}\right)}{2\left(m_{2}-m_{1}\right)}$$

where $x_{1}$ and $y_{1}$ are the x and y coordinates of point 1 in the axial plane, respectively; $m_{1}$ is the slope of the line connecting point 1 and point 2; and $m_{2}$ is the slope of the line connecting point 2 and point 3, calculated as follows:

$$m_{1}=\frac{y_{2}-y_{1}}{x_{2}-x_{1}}\;m_{2}=\frac{y_{3}-y_{2}}{x_{3}-x_{2}}$$

The radius of curvature was eventually calculated:

$$R=\sqrt{{\left(x_{1}-x_{C}\right)}^{2}+{\left(y_{1}-y_{C}\right)}^{2}}$$

For each area of the femur (total, proximal, middle and distal) the variation in the radius of curvature between the baseline and the follow-up CT was calculated for each individual patient, both for the prosthetic (Formula (2a): $∆R_{P}$) and for the contralateral (Formula (2a): $∆R_{C}$) side, considered as a control group:

(2a) $$\Delta R_{P}=R_{P,post}-R_{P,pre}$$

(2b) $$\Delta R_{C}=R_{C,post}-R_{C,pre}$$

### 2.3. Statistical Analysis

Statistical analysis was performed in the Matlab^®^ environment. The significance threshold (*p*) was set at 0.05.

In order to test the robustness of the curvature estimation method operator, a second operator segmented the femur canal of all patients. The radii of curvature measured by the two operators were compared using the Wilcoxon signed-rank test.

Differences in the SFB between the prosthetic and the control femurs in the first CT scan for each patient were calculated using the Wilcoxon test for paired data. The Wilcoxon test for paired data was also used to assess differences in the SFB of the two consecutive CT scans, comparing the prosthetic and the contralateral sides.

## 3. Results

Out of 4511 CT scans, 20 CT scans of 10 patients were eligible for the study: all patients underwent THA for hip osteoarthritis. Descriptive statistics are shown in Table 1.

Of the 10 patients under study, 8 patients were implanted with anatomic cementless stems (Apta stem, AdlerOrtho©, Milan, Italy; stem type 6, according to Khanuja), and 2 patients with conical tapered cementless stems (Wagner Cone stem, Zimmer Biomet©, Warsaw, IN, USA; stem type 3b, according to Khanuja) [12].

CT measurements of treated and untreated sides from the first CT scan (anatomical native features), and measurements of treated sides from the second CT scan are reported in Table 2 and Table 3, respectively.

Between the two operators, there were no statistically significant differences in proximal (*p* = 0.386) and middle (*p* = 0.522) femoral segment measurements. A statistically significant difference was found in the distal (*p* = 0.013) and total (*p* = 0.007) radii of curvature between the two operators’ measurements. For this reason, the results relating to the proximal and middle segments were statistically evaluated. Results obtained on the distal segment and on the total radius of curvature were excluded as they were considered unreliable.

The radii of curvature, preliminarily measured in the sagittal plane of treated and native femurs from the first CT scan, were not statistically significantly different (*p* > 0.05).

**Figure 4:**
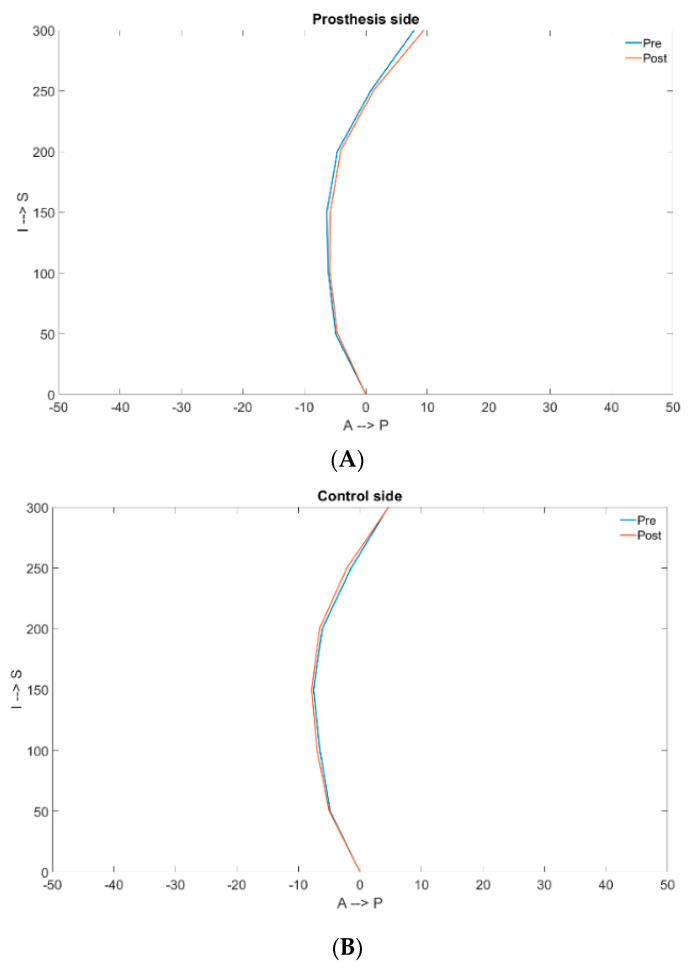
Comparison of the pre- and post-surgical femur curvatures in a prosthetic (**A**) and a native (**B**) side.

The difference in variations of the radius of curvature in the sagittal plane between the prosthetic and the control group was statistically significant for the proximal femoral segment (*p* = 0.004), but not for the mid-femoral segment (*p* = 0.56) (Table 4, Figure 4).

## 4. Discussion

We retrospectively analyzed 20 CT scans of 10 patients who underwent two consecutive CT scans (extending from the fourth lumbar vertebra to the tibial plateaus bilaterally), performed before THA and at least 3 years after THA (6.5 years after surgery). The hypothesis of modification in the radius of femoral curvature in the sagittal plane after implantation of a cementless femoral stem in comparison to the untreated side was confirmed: the sagittal radius of curvature significantly changed in the proximal segment of the THA side with respect to the untreated side.

According to our knowledge, this is the first study in the literature reporting the effect of primary cementless stems on SFB, comparing the outcomes to the untreated side. However, the study has some limitations. The main limitation is related to the modest number of patients under examination: this strict selection was due to complex measurements, the need for consecutive CT scans after a timespan of at least three years (to obviate ongoing modifications), and the need for a control group (to limit chronological changes involving femoral morphology). Another limitation is linked to the type of database from which patients were selected; since these CT scans were performed mostly for pre-operative planning purposes, some patients exhibited some types of aberrant anatomies or dysmorphisms. For this reason, the radii of curvature were sometimes far from the averages of the general population [13,14,15,16]. For this reason, the data were analyzed for variation in the two consecutive CTs and compared to the contralateral untreated side (not subjected to femoral implantation), after preliminary analysis of the first CTs. Finally, the search for the centroid of the medullary canal in sections with THA may be affected by artifacts related to the presence of the prosthesis, and this may have affected the measurements.

Several clinical studies have shown in detail the anatomy of the femur and its curvatures [2,16,17,18]; these data have often been used in the development of orthopedic implants, such as intramedullary nails and prosthetic designs, and in their implantation as well [6,13].

We wondered whether the loss of bone density in association with the modification of the modulus of elasticity of the proximal femur that occurs after implantation of cementless femoral stems [19,20] could change sagittal femoral bowing over time. Two observers were involved, adopting semiautomated measurement techniques on two consecutive CT scans. Measurements obtained by the second observer showed a non-statistically significant difference from the first observer in the proximal and middle radius of curvature, while a statistically significant difference was noted in the distal and total radius of curvature. These differences were due, in our opinion, to the complex anatomy of the distal femur, where, unlike the middle and proximal segments, it is difficult to identify the center of the medullary canal. Similarly, measurement of the overall radius of curvature was affected by that of the distal segment. Due to this limitation, this study cannot provide practical data about ipsilateral total knee arthroplasty after THA and complex stem revisions in Paprosky 4 defects.

The case series showed a statistically significant rate of change in the proximal sagittal radius of curvature (*p* = 0.004) after at least 3 years from cementless THA compared to the contralateral untreated side used as a control. No differences could be observed in the middle segment. This finding demonstrated that a cementless stem increases the SFB in the sole proximal segment, and this change is substantially independent from the variations induced by the aging process (as shown by the untreated side).

Thus, it is likely that the cementless femoral stem re-distributes the forces on the femur, leading to a new conformation of the proximal femur over time. It should be noted that only two types of conventional length stems were implanted, both with extensive porous coatings. If SFB changes may occur with single wedge stems or short femoral components, with only proximal porous coating, it becomes a matter of debate, and requires additional studies. However, these changes in SFB may have some practical implications. A direct impact of native femoral bowing on the range of motion of the replaced hip was observed by Akiyama et al.; the authors noted that the anterior bowing decreased flexion and internal rotation at 90° of flexion, possibly requiring some adjustments of cup positioning [21]. It is likely that chronological changes in SFB should be considered an additional factor influencing combined anteversion, posing further challenge to the definition of a “safe zone”. Secondarily, SFB changes may influence the possible femoral revision strategy. In cases of considerable femoral bowing, three possible options can be considered, according to the amount of bone loss and the revision setting: a primary conventional stem, a curved revision stem, or a conical tapered stem. However, in cases of relevant SFB, primary stabilization of a conical tapered revision stem without femoral osteotomy may be achieved through a three-point fixation. However, this is a suboptimal solution; thus, a femoral osteotomy (or a transfemoral approach) may be needed, adding some additional morbidity to the procedure [22]. At the same time, there is a chance that even a curved revision stem may require a femoral osteotomy for severe bowing. Thus, pre-operative detection of excessive SFB may improve pre-operative planning and influence stem selection and surgical approach. Moreover, it is of relevance for new revision stem development.

In summary, a primary cementless stem may impose consistent variation of SFB in the proximal part of the femur. This finding has many practical implications, from the study of impingement and combined anteversion in the primary THA, to the revision setting, in terms of selection of the appropriate stem and surgical approach. Proper planning can lead to a better approach to revision total hip replacements. This is of fundamental importance for those who deal with prosthetic revision surgery on a daily basis.

Knowing the variations on the sagittal plane, particularly in the proximal section of the femur, compared to the initial position of the first implant, can help surgeons in choosing the most suitable revision stem, considering the compression forces and tension that have acted up to that moment, and then adapting to them. Larger studies, with the involvement of different types of stems (single wedge and short stems), may give a more reliable perspective about the diffusion and the magnitude of SFB variations.

## Figures and Tables

**Table 1:** Descriptive statistics.

	Population under Study
**Patients**	10
**Average age at surgery (y)** ***(Min–max)***	54.3 *(32–73)*
**FU: Average time between surgery and second CT scan (y)** ***(Min–max)***	6.5 *(3–12.5)*
**Operated femur side:** **Right** **Left**	4 6
**Sex:** **F (female** **M (male)**	7 3
**Stem type:** **Anatomic** **Conical**	8 2
(y) = years	

**Table 2:** Native anatomical morphology of the first CT scans.

	Untreated Hip (Mean Value ± SD)	Treated hip (Mean Value ± SD)
Center of rotation height (mm)	20.3 (±1.6)	17.8 (±2.2)
Neck–shaft angle	131.5° (±11.9°)	142.7° (±12.7°)
Femoral offset (mm)	39.6 (±7.7)	36.1 (±7.2)
Acetabular offset (mm)	32.5 (±6.3)	30.1 (±4.6)
Femural anteversion	13.8° (±10.3°)	11.3° (±12.1°)
Acetabular anteversion	17.6° (±6°)	12.7° (±9.2°)
Leg-length discrepancy (mm)	24.4 (±8.6)	
Isthmus position (mm)	180.1 (±6.6)	176.4 (±5.9)
Medio-lateral femoral diameters at the isthmus (mm)	9.9 (±2)	12.3 (±1.1)
Anterior–posterior femoral diameters at the isthmus (mm)	11.7 (±2.2)	13.6 (±2.1)
Medio-lateral femoral diameters two centimeters below the lesser trochanter (mm)	15 (±3.4)	19.3 (±4.4)
Anterior–posterior femoral diameters two centimeters below the lesser trochanter (mm)	19 (±4.4)	22.8 (±1.8)
Medio-lateral femoral diameters two centimeters above the lesser trochanter (mm)	40.7 (±8.2)	47.1 (±8.5)
Anterior–posterior femoral diameters two centimeters above the lesser trochanter (mm)	29.9 (±3)	32.1 (±3.2)
Medio-lateral canal flares	3.86 (±0.9)	3.82 (±0.7)
Postero-lateral canal flares	3.05 (±0.7)	2.4 (±0.4)

**Table 3:** Treated cohort hip features (second CT scan).

Femoral anteversion	2.45° (±13.2°)
Acetabular anteversion	14.7° (±7.6°)
Femoral offset (mm)	37.9 (±13.8)
Global offset (mm)	66 (±6.3)
Sagittal tilt	3.29° (±2.08°)
Coronal tilt	2.08° (±1.36°)

**Table 4:** Difference in the variations in radius of curvature between prosthetic and control femurs in the sagittal plane.

Wilcoxon’s Test	Groups
	Total
(p) R* proximal sagittal curvature	0.004
(p) R* midsagittal curvature	0.55

## Data Availability

Data are contained within the article.
